# Cancer Associated Fibroblasts - An Impediment to Effective Anti-Cancer T Cell Immunity

**DOI:** 10.3389/fimmu.2022.887380

**Published:** 2022-04-11

**Authors:** Lilian Koppensteiner, Layla Mathieson, Richard A. O’Connor, Ahsan R. Akram

**Affiliations:** ^1^ Centre for Inflammation Research, Queen’s Medical Research Institute, University of Edinburgh, Edinburgh, United Kingdom; ^2^ Cancer Research UK Edinburgh Centre, Institute of Genetics and Cancer, The University of Edinburgh, Edinburgh, United Kingdom

**Keywords:** cancer-associated fibroblast (CAF), T cell exhaustion, targeting CAFs, mechanisms of immune evasion, tumour microenvironment

## Abstract

The presence of functionally efficient cytotoxic T lymphocytes (CTL) in the Tumour nest is crucial in mediating a successful immune response to cancer. The detection and elimination of cancer cells by CTL can be impaired by cancer-mediated immune evasion. In recent years, it has become increasingly clear that not only neoplastic cells themselves, but also cells of the tumour microenvironment (TME) exert immunosuppressive functions and thereby play an integral part in the immune escape of cancer. The most abundant stromal cells of the TME, cancer associated fibroblasts (CAFs), promote tumour progression *via* multiple pathways and play a role in dampening the immune response to cancer. Recent research indicates that T cells react to CAF signalling and establish bidirectional crosstalk that plays a significant role in the tumour immune response. This review discusses the various mechanisms by which the CAF/T cell crosstalk may impede anti-cancer immunity.

## Introduction

The tumour stroma plays a critical role in shaping the immune landscape in cancer. The most abundant stromal cells of the TME are cancer- associated fibroblasts (CAFs). Fibroblasts are typically activated during wound healing and revert to their quiescent state after exerting their function. However, in cancer, they remain perpetually activated by a number of factors including the presence of cancer cells, as indicated by their expression of activation markers [e.g., α smooth muscle actin (αSMA), fibroblast activation protein (FAP)] and promote tumour progression *via* multiple pathways. CAFs secrete angiogenic factors [e.g. vascular endothelial growth factor (VEGF)], factors degrading the basal membrane [matrix metallopeptidases (MMPs)], which promotes metastasis, and even alter their metabolic profile to produce energy metabolites (lactate, pyruvate) useful for cancer cells (“reverse Warburg effect”) (1). Furthermore, a growing body of research shows that CAFs are implicated in cancer immunotherapy failure across cancer types, and inhibiting CAFs revives the antitumour immune response in preclinical studies (2, 3).

CAFs employ immunosuppressive functions that involve various immune cells and stages of antitumoral immunity. In this review, we aim to establish a framework to understand the part CAFs play in inhibiting an efficient T cell response. An efficient T cell response relies on a number of orchestrated steps involving many cell types in the TME and results in cancer cell killing by cytotoxic T cells. In an ideal scenario, antigen presenting cells (APCs) present tumour antigen to naive T cells within tumour draining lymph nodes, inciting their activation, differentiation into cytotoxic T cells and travel to the tumour site. Here supported by a Th-1 mediated response of CD4+ T helper cells, primed activated cytotoxic T cells recognize their cognate antigen on the surface of cancer cells leading to clonal expansion of tumour specific effector T cells. This is followed by secretion of cytotoxic granules containing perforin and granzymes and killing of target cells in an antigen directed manner (4, 5). Successful T cell infiltration and the immune landscape of the TME are determining factors in the failure or success of the anti-cancer response.

## How Can CAFs Be Classified?

Various cell types can give rise to CAFs if exposed to environmental triggers such as TGF-β, platelet derived growth factor (PDGF) and fibroblast growth factor (FGF) (6). The absence of lineage markers such as cytokeratin and CD31 helps identify fibroblasts and while there is no specific marker to stringently differentiate CAFs from fibroblasts in adjacent non-cancerous tissue, CAFs typically show enhanced expression of surface markers αSMA, fibroblast specific protein (FSP), PDGF and FAP (6).

It is now widely recognised that CAFs display high heterogeneity (7, 8). Known activation markers of CAFs have been investigated by multiple groups and found to display difference expression levels within different CAFs, which have been characterised as subtypes (9–11). Common markers used to distinguish these subtypes include FAP, CD29, αSMA, podoplanin (PDPN) and platelet-derived growth factor receptor beta (PDGFRβ). The subtypes are defined by the expression levels of these markers, as no single specific marker for CAFs exists (12). Studies have defined subtypes of CAFs present in cancers such as breast cancer, ovarian cancer and pancreatic cancer. The markers used to define these CAF subsets are shown in **Table 1**.

**Table 1:** Definitions of CAF subsets identified in breast, ovarian and pancreatic cancer showing the differences in classifications.

CAF Subset	Origin	Markers	Reference
CAF-S1	Breast cancer	FAP^High^, CD29^Med-High^, αSMA^High^, PDPN^High^, PDGFRβ^High^	Costa et al. (10)
CAF-S2		FAP^Neg^, CD29^Low^, αSMA^Neg-Low^, PDPN^Low^, PDGFRβ^Low^	
CAF-S3		FAP^Neg-Low^, CD29^Med^, αSMA^Neg-Low^, PDPN^Low^, PDGFRβ^Low-Med^	
CAF-S4		FAP^Low-Med^, CD29^High^, αSMA^High^, PDPN^Low^, PDGFRβ^Med^	
CAF-S1	Ovarian Cancer	CD29^Med-High^, FAP^High^, αSMA^Med-High^, FSP1^Med-High^, PDGFRβ^Med-High^, CAV1^Low^	Kanzaki and Pietras (8)
CAF-S2		CD29^Low^, FAP^Neg^, αSMA^Neg-Low^, FSP-1^Neg-Low^, PDGFRβ^Neg-Low^, CAV1^Neg^	
CAF-S3		CD29^Med^, FAP^Low^, αSMA^Low^, FSP1^Med-High^, PDGFRβ^Med^, CAV1^Neg-Low^	
CAF-S4		CD29^High^, FAP^Low^, αSMA^High^, FSP1^High^, PDGFRβ^Med-High^, CAV1^Neg-Low^	
myCAF	Pancreatic Cancer	αSMA^High^, IL-6^Low^	Öhlund et al. (11)
iCAF		αSMA^Low^, IL-6^High^	

The most notable subsets investigated are the CAF-S1 and CAF-S4 as they have been found to be associated with cancer cell invasion and poor prognosis in breast cancer (10, 13). CAF-S1 were found to promote cancer cell migration and epithelial-mesenchymal transition (EMT) by the CXCL12 and TGFβ pathways whereas CAF-S4 were found to promote cancer cell invasion, particularly in three-dimensional models through NOTCH signalling. These more aggressive subsets of CAFs show the utility of being able to identify the level of heterogeneity within patient CAFs to determine which treatment mechanisms will be effective and to predict prognosis.

Studies have shown that these different CAF subtypes also play different roles in immunosuppression, and these roles would be a consideration when designing new therapies (7, 14). Illustrating the magnitude of differences between CAF subtypes, Costa et al. show CAF S1 (CD29^Med^ FAP^Hi^ FSP1^Low-Hi^ αSMA^Hi^ PDGFRb^Med-Hi^ CAV1^Low^) promotes the activation and differentiation of CD25^+^ T cells to FoxP3^+^ Treg, whereas CAF S4 (CD29^Hi^ FAP^Neg^ FSP1^Low-Med^ αSMA^Hi^ PDGFRb^Low-Med^ CAV1^Neg-Low^) does not, even though both are αSMA^high^ and could therefore be classified as CAFs. Significant differences in their inhibitory capacity were also seen regarding cytokine production and migration of T cells (10). Therefore, merely identifying CAFs by their expression of e.g., αSMA, might include CAF subtypes with very different functions in the TME. In fact, a more recent study further divided Costa et al.’s CAF S1 subtype into 8 clusters based on single cell RNA sequencing of 19000 CAF S1 breast cancer fibroblasts. Two of those clusters were associated with immunomodulation, one of which also promoted FoxP3^+^ T cell frequency within CD4^+^CD25^+^ T cells whereas others did not, further highlighting the heterogeneity of the population (15).

In some tumours, such as neuroblastoma, certain mesenchymal stromal cells also harbour immunomodulatory properties, and while these also express the potential fibroblast marker CD90 it is not known whether they align with a specific CAF subtype found in other types of cancer (16).

CAF heterogeneity poses a challenge in CAF research, as separate studies use different classifications for CAF subsets illustrating the need for a unified approach. Considering different CAF classifications in the current literature, we do not restrict this review to a specific subcategory of CAF.

## How Do CAFs Restrict T Cell Migration?

### ECM and Stromal Density

The immune landscape of cancer is classified in three distinct immunophenotypes. Inflamed, “hot” (1), tumours are characterised by T cell infiltration and associated with enhanced response to checkpoint inhibition (17). It is well understood that the infiltration of cytotoxic T cells into the tumour nest is a prerequisite for T cell mediated killing of cancer cells (18). As a result, “cold” tumours are associated with poor response to checkpoint inhibition and a failed immune response. Here, CD8^+^ T cells are either absent [“deserted” (2)] or do not effectively infiltrate the tumour islands, as they are either restricted to the invasive margins or the stromal regions of the tumour, unable to be in physical contact with cancer cells [“excluded” (3)]. Factors determining the immunophenotype of a tumour include tumour mutational burden (TMB) and MHCI expression and recent research indicates a crucial role of CAFs (19).

**Figure 1:**
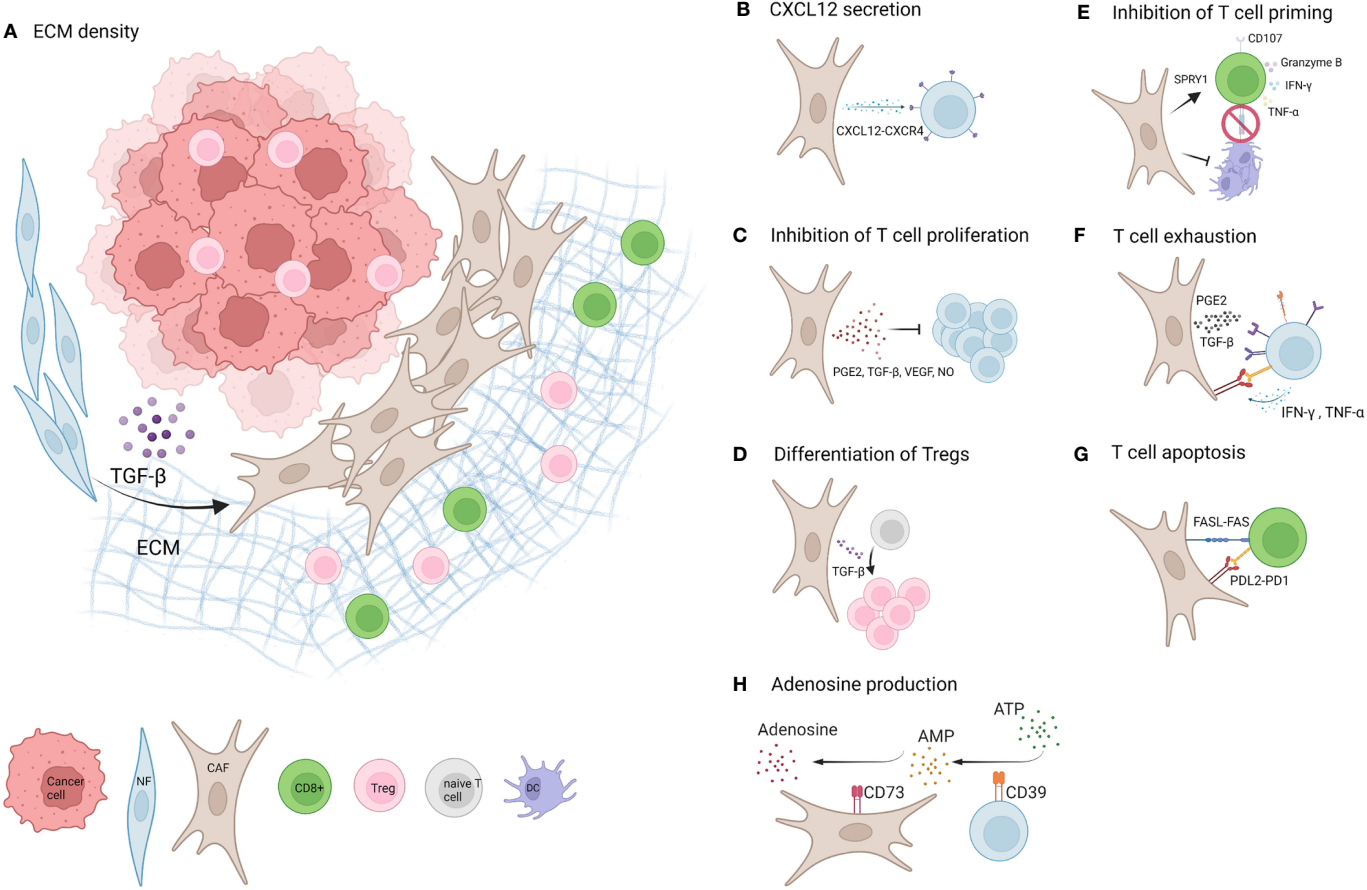
Mechanisms of CAF mediated T cell inhibition. **(A)** Factors including TGF-β and ECM promote the development of CAFs in the TME of solid tumours. A dense network of CAF secreted ECM restricts T cell mobility and entry of CD8^+^ T cells to the tumour nest (1). **(B)** CXCL12 secreted by CAFs binds to CXCR4 on T cells and likely contributes to T cell restriction to the tumour stroma (20–22). **(C)** CAFs limit T cell proliferation by factors including PGE2, TGF-β, VEGF and NO (23–27). **(D)** One study suggests CAF secreted TGF-β promotes differentiation of naive CD4^+^ T cells into FOXP3^+^Tregs (28). **(E)** CAFs inhibit dendritic cell (DC) differentiation and maturation and thereby limit T cell priming (7, 29). Additionally CAFs upregulate SPRY1 in T cells associated with reduced activation and downregulate CD107, and cause a reduction in secretion of Granzyme B and TNF-α. Opposing studies report an increase or decrease of IFN-γ secretion by T cells (7, 30–32). **(F)** CAFs upregulate exhaustion markers PD1, Lag3, TIGIT, Tim3and CD39 on T cells (*via* e.g. PGE2 and TGF-β) and T cells upregulate PD-L1 and PD-L2 expression on CAFs *via* IFN-γ and TNF-α (15, 22, 23, 33–36). **(G)** CAFs promote apoptosis of T cells *via* high levels of FASL and PDL2 expression and upregulation of FAS and PD1 on T cells (35). **(H)** CAFs upregulate CD39 on T cells and T cell secreted IFN-γ and TNF-α upregulate CD73 expression on CAFs, which could potentially increase the production of immunosuppressive Adenosine (37). Created with BioRender.com.

Main mechanisms of CAF mediated effects on T cells are depicted in **Figure 1**. One mechanism of CAF mediated immunotherapy failure is the CAF-induced increase in ECM density. Firstly, this results in reduced drug penetration of the tumour tissue (1). Secondly, the tight matrix and increased interstitial fluid pressure in CAF rich stroma can also promote T cell exclusion from the tumour nest. Salmon et al. used live cell imaging to investigate the localisation and migration of T cells in tumours. Fluorescently dyed freshly isolated TILs were added on top of human lung tumour slices *in vitro*. Added TILs accumulated at 5x higher numbers in the tumour stroma compared to tumour islets and travelled along linear tracks parallel to stromal fibres. They further showed that T cell counts were negatively correlated with ECM density and that fibronectin rich regions, such as areas immediately surrounding tumour islets, inhibited T cell motility (38). Interestingly, Blair et al. report that pharmacologically degrading stromal hyaluronan resulted in an increase in effector memory CD8^+^ TIL and improved antitumour immunity in a murine model of pancreatic ductal adenocarcinoma (39).

Multiple studies have reported reduced CTL infiltration in CAF rich tumours compared to their CAF low counterparts (33, 40). For example, Kato et al. report CD8^+^ T cells to be located in peritumoral rather than intratumoral tissue in CAF-rich oesophageal cancer, while in CAF-low tumours, CD8^+^ T cells were found to be distributed across both sites (40).

CAFs expressing high levels of FAP and αSMA are prominently implicated in CD8^+^ T cell exclusion. In line with Salmons study, Gorchs et al. describe dense stroma surrounding tumour nests in pancreatic cancer and report that these areas express αSMA (23). Gene analysis of CAFs and normal fibroblasts (NF) in ovarian cancer showed nine differentially expressed genes linked to CD8^+^ T cell infiltration. The upstream regulator of three of the genes, preselin1 (PS1), co-localises with CAF activation markers FAP and αSMA and was associated with low TIL counts. PS1 silencing reduced the expression of FAP and αSMA on CAFs and reduced tumour burden in an ovarian tumour mouse model *via* increased CTL infiltration, indicating that PS1 has a significant role in CAF mediated T cell exclusion from the tumour nest, likely upstream *via* promoting CAF activation (41). Using a whole tumour cell vaccine genetically modified to express FAP in a murine model of melanoma and lung cancer, resulted in reduced tumour growth and prolonged survival that was dependent on enhanced CD8^+^ TIL infiltration. This effect was significantly higher than that of unmodified whole tumour cell vaccine and was attributed to directly inhibiting CAFs, as it resulted in reduced FAP – and collagen type I expression in these tumours (2).

Ford et al. investigated how CAFs confer immune checkpoint inhibition (ICI) resistance using CAF rich murine tumour models. They previously reported that the downstream target of TGF-β1, NADPH oxidase 4 (NOX4), which generates reactive oxygen species (ROS), can regulate fibroblast differentiation into myofibroblasts (42). Using single cell RNA sequencing, NOX4 expression was correlated to CAF markers including FAP, Thy1, decorin, and collagen type I and VI (43). Targeting NOX4 in murine models of CAF rich tumours (either by silencing or pharmacologically inhibition) suppressed the TGF-β mediated differentiation into myofibroblasts and additionally downregulated functional markers including αSMA and collagen 1 of fully differentiated CAFs resulting in a more “quiescent” state and a rescued CD8^+^ TIL response by redistribution of CD8^+^ T cells into the tumour (33). These studies suggest that the activation of CAFs and the resulting increased synthesis of matrix components such as hyaluronan are implicated in CTL exclusion. Pharmacologically targeting CAFs to reduce ECM density could improve T cell trafficking to the tumour nest which could increase efficacy of checkpoint inhibition therapy as well as improving drug penetration of agents that directly target cancer cells.

Fibroblastic reticular cells (FRCs) employ chemotactic strategies to modulate T cell trafficking in the healthy lymph node by expression of lymphocyte attractants CCL19 and CCL21 which bind to CCR7 on naïve T cells (34). Similarly, in cancer, aside from the physical restrictions of dense ECM, it has come to light that CAFs secrete factors which directly influence T cell migration and function in the TME. The best characterised of these mechanisms is the CXCL12-CXCR4 chemokine axis which contributes to CTL exclusion. CXCL12, produced by FAP^+^CAFs in the TME binds to its receptor CXCR4, expressed by T cells, thereby trapping TIL in the tumour stroma and restricting their access to tumour areas containing cancer cells (20). Feig et al. demonstrated the significance of this pathway in a pancreatic ductal adenocarcinoma mouse model by showing that inhibition of CXCR4 caused a redistribution of T cells within the tumour tissue, improved CTL activity and decelerated tumour growth (21). Notably first clinical data shows CXCR4 inhibition in human pancreatic ductal adenocarcinoma also increased CD8^+^ T cell infiltration into the tumour (44).

Another factor attenuating CD8 T cell infiltration is Fibroblast growth factor-β (FGF2) a cytokine released by cancer cells that activates quiescent fibroblasts and upregulates CAF marker expression like αSMA and FAP (45). In a mouse model of pulmonary metastasis inhibition of FGF Receptor signalling resulted in a dose dependent increase in CD8 T cell infiltration and significantly delayed tumour growth. Mechanistically, gene expression data of urothelial carcinoma cells treated with a different FGFR inhibitor revealed FGFR inhibited T cell chemoattractant CXCL16 and CD8 T cell infiltration and effects were attributed to inhibiting FGFR on cancer cells. CAFs were not investigated in this study but it could be hypothesised that the delay in tumour growth and increase in CD8^+^ T cell infiltration could be partly mediated by a negative regulation of CAFs *via* FGFR inhibition (46).

Production of TGF-β by CAFs also suppresses anti-tumour immunity. CAFs produce significantly more TGF-β than normal fibroblasts from non-cancerous tissue and TGF-β is particularly high in immunosuppressive PDPN^high^ CAFs (47, 48). Similarly, single cell sequencing of breast cancer fibroblasts, identified a subtype of CAFs expressing high levels of TGF-β which correlates with resistance to immunotherapy (15).

Recently, Desbois et al. demonstrated, using a combined IHC and transcriptome analysis approach of a large ovarian cancer cohort, that a key hallmark defining T cell excluding tumours is the upregulation of TGF-β and activated stroma. Mechanistically, the majority of TGF-β elicited changes in the transcriptional programme consists of ECM related genes, glycoproteins, and reactive stroma markers, reinforcing the idea that the main mechanism of T cell exclusion is ultimately the establishment of a physical barrier by activated stroma (49). Similarly, TGF-β plays a significant role in urothelial cancer, where blocking TGF-β allowed T cell entry to the centre of the tumour, followed by tumour regression (50). Furthermore, Desbois et al. described that while immune deserted tumours had a slightly lower neoantigen load, they did not differ from infiltrated tumours in neoantigen load or TMB. Rather the main difference, was a downregulation of antigen presenting genes and low MHC-I expression mainly in the tumour compartment. While deserted tumours had overall low MHC-I and infiltrated tumours showed strong homogenous MHC-I on tumour cells, it seems T cells were trapped in the MHC-I expressing stroma (49). While cancer cells downregulate MHC-I to avoid CTL killing, Phipps et al. have seen an upregulation of MHC-I on CAFs in response to IFN-γ and in line with this we have seen the same effect induced by activated T cells (37, 51). It could be postulated that this is an additional mechanism of retaining passing T cells in FAP^+^ CAF rich stroma on their way to the tumour nest. Furthermore, gene expression analysis of TGF-β activated fibroblasts shows upregulation of genes encoding immunomodulatory cytokines IL11, TNF-AIP6 and IL-6 (49). CAFs produce significantly more IL-6 than normal fibroblasts and thereby promote epithelial to mesenchymal transition in NSCLC (52). Interactions of CAFs with cancer cells additionally results in a dramatic increase in IL-6 production *in vitro* (53). Notably, FRC derived IL-6 is known to be able to affect the fate of T cells during T cell priming in the lymph node suggesting this as a possible mode of T cell modulation by CAFs in cancer (34). Interestingly, high IL-6 secretion is significantly associated with a particularly immunosuppressive FAP^+^ CAF phenotype (CAF S1) in breast cancer and a dominant feature of an inflammatory CAF phenotype in pancreatic ductal adenocarcinoma (10, 11). Single cell RNA sequencing of this inflammatory CAF subtype showed upregulation of other inflammatory pathways including IFN-γ, TNF and NFKβ. Notably hyaluronan synthases as well as matrix proteins were specific to this subset, suggesting this subset is active in producing dense extracellular matrix (54). Ohno et al. report that IL-6 deficient mice showed significantly decreased tumour growth of colon cancer compared to wildtype mice. This observation co-occurred with increased numbers of IFN-γ producing T cells, increased PDL1 and MHC1 expression on cancer cells and was dependent on CD8^+^ T cells (55). Using a colon cancer mouse model, Kato et al. showed that co-transferring fibroblasts together with cancer cells resulted in slightly increased tumour growth in immunodeficient nude mice, and that this effect was more pronounced in immunocompetent mice, suggesting CAFs support tumour growth *via* modulating the immune response. Notably, they established that CD8^+^T cell exclusion caused by CAFs was dependent on IL-6, as tumours regularly injected with IL-6 mirrored the effects of CAFs and IL-6 blockade caused a significant shift in the TIL population from FoxP3^+^ to CD8^+^ T cells (40). Additionally, blocking IL-6 together with PD1-PDL1 blockade caused a significantly improved T cell response (56). Similarly, co-administering TGF-β antibody and anti-PDL1 in a mouse model of urothelial cancer caused a reduction in TGF-β signalling in stroma and allowed T cell infiltration into the tumour centre, suggesting that these two cytokines secreted by CAFs might offer targets to relieve the immunosuppressive effects of CAFs on immune cells and promote efficacy of ICI (50).

Multiple studies have shown that CAF high tumours have low CD8 T cell infiltration, while Treg infiltration is actually increased in these tumours indicating that active stroma may affect cytotoxic T cells and Treg differently. A recent *in vitro* study modelling matrix stiffness in a 3D culture system, offers some insight into possible underlying mechanisms, and reports that the viability of CD4^+^ T cells exceeded that of CD8^+^ T cells in ECM with high rigidity, which could indicate that CD8^+^ T cells are more sensitive to mechanical pressure (57). Further studies are needed to understand the specific mechanisms by which CAFs selectively exclude CTL and promote Treg.

## What Happens to T Cells in the Stroma?

### CAFs Inhibit T Cell Proliferation

As T cells are sequestered in the tumour stroma, their phenotype and function are directly affected by the influence of surrounding CAFs. Multiple *in vitro* studies show that the presence of CAFs significantly reduces the proliferation of both CD4^+^ and CD8^+^ T cells in a contact-independent manner, suggesting this as a possible explanation for reduced TIL frequencies in CAF high tumours (23–27). In fact, using a pancreatic cancer cell line, Gorchs et al. demonstrate that CAFs have higher inhibitory potential on T cell proliferation than cancer cells do (23). In terms of the underlying mechanisms, multiple factors have been reported to mediate this inhibition, such as CAF-derived prostaglandin E2 (23). and AKT3, a protein kinase with a role in immunosuppressive activity of CAFs (58). Having established that PS1 is an upstream regulator of genes differentially expressed in CAFs compared to normal fibroblasts and a factor promoting CAF expression of the activation markers FAP and αSMA, Zhang et al. report that silencing PS1 reversed the anti-proliferative effects of CAFs on T cells (41). Takahashi et al. investigated PD-L1 and PD-L2, co-inhibitory ligands expressed by a subset of CAFs which inhibit T cell activation and - function *via* PD-1 binding. The authors report that blocking PD-L1 and PD-L2 normalised T cell proliferation and additionally, a similar effect was shown by neutralising CAF-secreted VEGF and TGF-β, possibly due to a loss of the CAF stimulating effect of these signals (24). Conversely, Gorchs et al. see no significant changes in proliferation following TGF-β blocking (23). It is important to consider that study designs varied in terms of cancer type and model and that CAFs comprise a particularly heterogenous population of cells and without further discrimination of their specific phenotype, results are likely to vary. Cremasco et al. looked into nitric oxide (NO) production by CAFs as a mechanism of inhibiting T cell proliferation in cancer, as this has been found to be a mechanism employed by FRCs after sensing T cell secreted IFN-γ and TNF-α to limit their proliferation in the healthy lymph node. Underlining the functional heterogeneity of CAFs, they report a similar mechanism in breast cancer where PDPN^+^ CAFs significantly inhibited the proliferation of CD4^+^ and CD8^+^ T cells in co-culture *via* production of NO while PDPN^-^ CAFs do not (34, 59). More drastically, CAFs can limit the cytotoxic T cell pool by inducing apoptosis in CD8 T cells *via* FAS ligand and PD-L2 engagement (35).

## Do CAFs Truly Drive Regulatory T Cell Differentiation?

While restricting CD8^+^ T cell infiltration, recent research indicates that specific CAF subtypes can selectively attract and retain CD4^+^ CD25^+^ T cells (15). Tumours high in FAP^+^CAFs are positively associated with an increase in FoxP3^+^ Treg infiltration (40, 60–62). Notably, Givel et al. report that mesenchymal high-grade serous ovarian cancer which is associated with poor survival showed high stromal density of fibroblasts and an enrichment for the CAF-S1 subtype, which was associated with a significant increase in FOXP3^+^ cells compared to tumours enriched with CAF-S4 fibroblasts. This was attributed to high expression of the CXCL12β isoform of this subtype, as *in vitro* functional experiments showed an increased migration of CD4^+^CD25^+^ T cells, but not CD4^+^CD25^-^ T cells in the presence of CAF-S1 that was dependent on CXCL12β. In addition to the enhanced attraction of CD4^+^CD25^+^ T cells, co-culture of CAF-S1 fibroblasts and CD4^+^CD25^+^ T cells demonstrated an increase in CD25^+^FOXP3^+^ T cells and enhanced their survival in a contact dependent manner (9) Similarly, in breast cancer, the above mentioned immunosuppressive CAF S1 subtype correlates with an increase in FOXP3^+^ regulatory T cells. *In vitro* co-culture of CAF S1 and T cells revealed a shift towards FOXP3^+^ Tregs that was dependent on immune checkpoint molecules B7H3, CD73 as well as DPP4, a membrane bound enzyme closely related to FAP that is known to cleave the effector T cell chemoattractant CXCL10 (10). Furthermore, multiple studies report an increased frequency of FOXP3^+^ cells after co-culture of PBMCs from healthy donors with CAFs compared to normal fibroblasts. This shift has previously been attributed to CAFs driving Treg differentiation, could however also result from differences in proliferation amongst FOXP3^+^ cells and other T cells or increased survival of existent Tregs rather than an induction of FOXP3 amongst naïve T cells (23, 24). Notably however, Kinoshita et al. exposed purified naïve conventional CD4^+^ T cells (CD4^+^25-CD45RA^+^) from healthy donor PBMCs to supernatant from CAFs from Treg-high lung tumours and did see a significant increase in FOXP3^+^ cells compared to supernatant from CAFs from Treg-low tumours that was mirrored by treatment with TGF-β suggesting true induction of Tregs *via* CAF secreted TGF-β (28). Furthermore, a murine fibroblast cell line transfected with FAP induced the differentiation of primary cultured murine splenocytes to CD4^+^CD25^+^T cells, however the frequency of FOXP3^+^ amongst this pool is not reported and mediating factors are unknown (63).

Notably, when exposing stimulated T cells to CAFs, differentiation into effector- (CD45RA^-^CCR7^-^) and central (CD45RA^-^CCR7^+^) – memory T cells amongst proliferating T cells is significantly reduced. Instead a larger pool of T cells remains in their naïve state (CD45RA^+^CCR7^+^) (23). In addition to possibly promoting regulatory T cells, TGF-β could also negatively affect cytotoxic T cell differentiation, as a recent study on oral squamous cell carcinoma illustrates the significance of TGF-β in attenuating the cell cycle of CTL, inhibiting their proliferation during effector phase as well as their differentiation into TEM, promoting apoptosis induction, and ultimately causing a decrease in the CD8^+^ T cell/Treg ratio (64). In line with this, inhibiting TGF-β in a mouse model of pancreatic cancer in combination with gemcitabine caused an increase in naïve Treg markers (CD62L, CCR7) and downregulation of markers associated with an effector/memory Treg phenotype. Additionally, inhibiting TGF-β caused a reduction of Treg-mediated suppression of CD8^+^ T cells. This suggests that TGF-β could promote the development of effector memory Tregs with suppressive activity against CTL, further highlighting the role of TGF-β in indirectly impairing CTL function in cancer (30). To conclude, CAFs could potentially mediate induction of Tregs and suppress memory T cells, however, current evidence is not conclusive.

### CAFs Attenuate T Cell Activation and Prevent Effective T Cell Priming

In the lymph node, FRCs upregulate immunostimulatory factors (ICOS ligand, CD40 and IL-6) in response to signals from activated T cells which enhances IL-2 and TNF-α production by activated CD8 T cells. Conversely, in cancer, some studies indicate that CAFs reduce CTL activation. The presence of melanoma CAFs during CD8^+^T cell activation reduces the percentage of cells exhibiting the early T cell activation marker CD69. Additionally, in PBMCs co-culture, CAFs promote an increase in cytokines typical for Tregs, such as IL-10 and TGF-β in line with their positive influence on Treg differentiation, and thereby add to the consequent anti-inflammatory environment. In fact, when pre-exposed to the CAF subtype CAF-S1, CD25^High^CD127^low^CD45RA^low^ T cells increased the ability to inhibit the proliferation of effector T cells. CAFs thereby equip Tregs with increased suppressive activity against effector T cells (10).

It has been shown that CAFs also affect other immune cells in the TME and mediate T cell suppression indirectly [reviewed (7)]. For example, DCs conditioned with supernatant of CAFs, induce a TH2 cytokine response from CD4^+^ T cells during co-culture (29). Additionally, CAFs have recently been shown to suppress DC differentiation, maturation and enhance CD11c^+^ inhibitory phenotypes ultimately inhibiting CD8^+^ T cell priming, likely *via* the WNT catenin signalling pathway (31) Furthermore, CAFs interact with tumour-associated macrophages (TAMs) in a reciprocal fashion, promoting the development of an M2 TAM phenotype with pro-tumour functions such as expression of PD1-ligands which ultimately impairs cytotoxic T cell function [reviewed (65, 66)]. Consequently, CAFs might reduce CTL activation directly as well as indirectly, either *via* modulation of the environment or through the modulation of their interaction with other immune cells.

A recent study in oesophageal cancer shines some light on how CAFs facilitate inhibition of T cell activation. In response to FGFR signalling by αSMA^+^ fibroblasts, T cells upregulate the FGF2 antagonist SPRY1. SPRY1 reduces NfKB, NFAT, Ras MAPK signalling and limits T cell activation. FGF2 causes a significant reduction of IFN-γ, TNF-α and granzyme B production by *in vitro* stimulated CD8 T cells and decreased their efficiency in killing target cancer cells (67). Similarly, a subset of FGF2^+^ CAFs that secrete WNT2 are correlated with a high ratio of Foxp3^+^CD4^+^ T cells/CD4^+^ T cells and show reduced ratio of IFN-γ producing CD8^+^ T cells (31). Additionally, using a mouse model of systemic infection, Shehata et al. have shown that T cells negative for SPRY1 have enhanced survival (32). Furthermore, compared to NF, CAFs express higher levels of FASL, and additionally upregulate FAS expression on TIL, leading to the suppression of CTL activity (35). Notably, research suggests that CAFs also mediate suppression of CTL cytotoxicity *via* decreasing the expression of CD107a as well as granzyme B in T cells (22, 23, 68). Similarly, low expression of overall CXCR4 in tissue slides of pancreatic cancer was significantly associated with increased granzyme A and perforin, marking cytotoxic capacity of CTL. This highlights potentially increased T cell cytotoxicity when they are not attracted and retained to the stroma *via* the CXCR4-CXCL12 axis (39). In line with this, Zhang et al. report that silencing PS1, which as indicated above reduces FAP and αSMA expression on CAFs, causes an increase in the activity of CTL, here shown in an increased IFN-γ release (41). Other studies also report reduced levels of TH1 cytokines in the presence of CAFs (60). Using a 3D scaffold *in vitro* model of breast cancer, Phan-Lai et al. observed CAF-mediated suppression of TNF-α release by tumour reactive T cells (69). Again, reduced CXCR4 gene expression was also correlated with improved IL-17a cytokine production by CD8^+^TIL, likely due to increased CTL infiltration, and increase IFN-γ levels amongst isolated CD8^+^TIL (39). A recent study by Li et al. showed TGF-β inhibition caused a temporary shift from myCAF, which were located tightly around the tumour islands to iCAF which were loosely connected interspersed. Inhibiting TGF-β in combination with gemcitabine, caused an increase in IFN-γ production by CD8^+^ T cells as well as increased T cell activation markers 4-1BB (CD137) and OX40 and markers of cytotoxicity (granzyme and perforin) (30).

However, while Nazareth et al. report a suppressive effect of CAFs on T cell activation in 3/8 NSCLC tumours, in the other five tumours, CAFs surprisingly produced IFN-γ, and induced the activation of T cells, which increased their response to TCR stimulation, a phenomenon that could be partially reversed by TGF-β (47). Interestingly, a study by Barnas et al. revealed that fibroblasts from lung tumours, non-cancerous lung tissue or even skin fibroblasts increased the secretion of IFN-γ and IL-17 by lung cancer TILs (70). This effect was only observed in the presence of activation stimuli and was in part mediated by a common CAF cytokine, IL-6, which was in turn increased by T cell conditioned media (70). The authors therefore attribute CAFs an immunostimulatory role in the TME. Similarly, we saw increased T cell production of IFN-γ in co-culture with CAFs. IFN-γ from T cells in turn, together with TNF-α, upregulates MHC I and - II on CAFs and in line with Barnas et al., increased CAF-production of IL-6 (37). While IFN-γ does activate cytotoxic T cells, it also negatively regulates TILs by upregulating PD1 ligand expression on CAFs and promoting production of IDO, (71). and therefore might not be purely immunostimulatory in this context. Additionally, T cells induce IL-27 secretion by CAFs, which again promotes PD1 ligand expression as well as inducing Tim3 expression and IL10 production by T cells (72). At the same time, IL-27 signalling supports granzyme B expression and proliferation of cytotoxic T cells, illustrating that CAFs may not rigidly act as either immunosuppressive- or stimulatory, but rather adjust their immunomodulatory activity depending on the extent of the T cell response (73).

### T Cells Find CAFs Tiring

As a final barrier to T cells that have overcome these hurdles, CAFs dampen the remaining CTL response to cancer cells by promoting the development of functionally exhausted CTL through the upregulation of co-IR expression. As a physiological regulatory mechanism to limit T cell cytotoxicity, T cell exhaustion is characterised by a progressive loss of effector function, expression of multiple co-IR and a common transcriptional and epigenetic program (36). Given that CAFs co-localise with PD-1^+^TIL in pancreatic cancer (23), researchers have investigated the effect of CAFs on the expression of other co-IR on TIL. Indeed, CAFs upregulate expression of Tim3, CTLA4, Lag-3 on both CD4^+^ and CD8^+^ TIL (23, 33, 35). Moreover, melanoma derived CAFs elicit TIGIT and BTLA expression in CD8^+^T lymphocytes, mediated *via* l-arginase (22). Deletion of stromal cells *via* targeting FAP using a vaccine significantly lowered PD-1 levels in preclinical models of melanoma (74). The underlying mechanisms are not fully understood; however, first reports show that Tim-3 and PD-1 upregulation was enabled by CAF derived PGE2 (23). Remarkably, in response to the T cell cytokine IFN-γ, CAFs react with an upregulation of PD-L1 and -2 expression (37, 47).

In a recent study investigating the modulation of phenotype and function of TIL by CAFs in NSCLC, in addition to upregulated PD-1 and TIM3, our group also observed an upregulated expression of CD39 on T cells when co-cultured with CAFs, that was mediated *via* TGFβ (37). Another co-IR, CTLA-4, is involved in adhesion and migration of T cells. In their murine models, Ford et al. saw an increase in CTLA-4 expression amongst CD8^+^TIL in CAF rich tumours using RNA sequencing and flow cytometry. Immunohistochemistry of human HNSCC tumours confirmed CTLA4 expression of on average 15.3% of excluded CD8^+^T cells. Blocking CTLA-4 in their murine lung tumour model increased CD8^+^T cell infiltration and reduced tumour growth (33). Taken together, these studies demonstrate that CAFs promote the progression of an exhausted phenotype of cytotoxic T cells.

## Do CAF- T Cell Interactions Affect Adenosine Levels?

An area of interest in CAF mediated immunosuppression, with a promising therapeutic target, is their role in the production of adenosine. Adenosine signals *via* P1 receptors on immune cells and exerts immunosuppressive functions. It is produced by hydrolysis of pro-inflammatory extracellular ATP (eATP), *via* the cell-surface enzyme CD39, into adenosine monophosphate (AMP), which is further converted into adenosine by CD73 (75). Expression of CD39 and P1 receptors is typically upregulated in response to tissue damage, tissue remodelling, hypoxia/oxidative stress and chronic inflammation as a means to protect the surrounding tissue from immune-mediated tissue damage. In recent years, it has become evident that CD39 is also expressed in the often chronic inflammatory, hypoxic environment of tumours. In cancer, adenosine has detrimental effects, as it promotes tumour growth and progression *via* suppressing the immune response. It enhances the immunosuppressive effects of tumour associated macrophages (TAM), myeloid derived suppressor cells (MDSC) and regulatory T cells while dampening immunostimulatory effects of neutrophils, NK cells and inhibits T cell priming by inhibiting the activation of dendritic cells (DCs) (75). Furthermore, reduction of available eATP and intracellular accumulation of cAMP in CTL significantly impairs their effector function.

We have recently shown that CAFs upregulate expression of CD39 on T cells and in turn, T cells upregulate CD73 expression on CAFs (37). They could thereby establish a feedforward loop to sustain adenosine in the TME, that is sensitive to the extent of T cell infiltration sustaining a local immunosuppressive environment. CD39 can also be expressed by CAFs, and upregulated, much like other CAF immunosuppressive factors (TGF-β, ARG, IDO, CXCL12, PGE2, PD-L1), during hypoxic stress (76). CAFs could therefore theoretically produce adenosine from ATP on their own, however, it has previously been shown that mesenchymal stromal cells are able to produce significantly more adenosine in the presence of activated CD39^+^ T cells than each cell type alone and additionally upregulate CD73 expression on CD4^+^ T cells (77). A recent study reported CD73 expression in the TME is mainly attributed to CAFs, as 75-90% of CD73 immunofluorescence staining of human colorectal cancer tissues was found on αSMA^+^cells (78). Interestingly, in the previously mentioned study by Costa et al., CD73 expression was particularly high in the highly immunosuppressive CAF S1 subset (10), which further implies that CD73 activity contributes to CAF mediated immunosuppression in the TME. Notably, Yu et al. demonstrate that the levels of adenosine in the TME regulates CD73 expression on CAFs *via* A2B receptors on CAFs in a feedforward loop amplifying immunosuppression in the presence of adenosine (78). CD73 expression levels are also affected by the cytokine milieu of the TME. A previous study has shown that IL-6 can upregulate CD73 on nasopharyngeal cancer cells (79), and Hu et al. reported similar observations for the effect of IL-6 on CD73 expression on γδ T cells (80). Importantly, they report that CD73^+^γδ T cells can in turn promote IL-6 production by CAFS. In line with this observation, Barnas et al. saw a synergistic increase in IL-6 production when activated T cells were co-cultured with CAFs derived from human NSCLC tumours (70). Furthermore, TGF-β, which is expressed more in CAFs compared to NF (24). has been shown to sustain CD73 expression on T cells (81). and promote IL-6 production by pericytes in NSCLC (82). IL-6 and IL27 are key factors in upregulating CD39 expression on TILs (83–85). IL-27 is prevalent in the TME and notably, our group has shown that the presence of TIL elicit IL-27 secretion by CAFs (37). And while we saw an upregulation of CD39 on T cells in the presence of CAFs, we found this was dependent on TGF-β. These studies suggest that in response to tumour infiltrating T cells, CAFs might promote the upregulation of factors needed for adenosine production in a complex interplay of IL-6, IL27, TGF-β and CD39 and CD73.

## Do CAFs Directly Interact With TIL?

As discussed above, CAF inhibitory effects can be mediated *via* soluble factors (23, 24, 26, 70). However, since TIL and CAFs colocalize in the tumour stroma, researchers have been curious as to whether they engage in direct cell-cell interactions. Indeed, CAFs have been shown to have the ability to uptake, process and present antigen *via* MHCII, thereby further increasing their potential to interact with TIL (54). Furthermore, using the antigen ovalbumin, Lakins et al. show CAFs were similarly efficient in processing antigen as FRCs, however unlike FRCs and normal fibroblasts, CAFs displayed delayed endosome mediated processing, like APCs, resulting in enhanced cross-presentation to T cells (35). Notably, CD74, involved in the formation of MHC II, is highly expressed on iCAF, a subcluster of CAF-S1, which is unlike the other subclusters ecm-myCAF and TGF-β-myCAF not associated with immune modulation (15). It remains to be seen whether the ability to present antigen *via* MHCII is a general CAF trait or if it is reserved for immunosuppressive CAFs. CAF-S1 additionally increase their contact with TIL by expressing the PD-1 co-IR ligands PD-L1 (47). and PD-L2 (10, 35, 47), the adhesion molecule JAM2, and OX40L (10), which have been confirmed to co-localise with CD4^+^CD25^+^ TIL in breast cancer (10). In fact, the presence of TIL increases the expression of MHC I and MHC II on CAFs, as well as their expression of PDL1 and PDL2, possibly in an effort to increase their interaction with TIL (37). Additionally, CAFs interact with other immune cell types which ultimately also affects TILs. Rodriguez recently uncovered a role of CAFs in the establishment of tertiary lymphoid structures (TLS), lymphoid formations in the tumour microenvironment that share similarities with secondary lymphoid organs and are correlated to enhanced survival and response to immunotherapies. *Via* secretion of CXCL13, CAFs drive expansion of tumour associated TLS by attracting B cells with the cognate receptor CXCR5. It is furthermore important to consider the stage of tumour progression as the immune landscape is highly dynamic and recent research shows three distinct functionally diverse stromal populations at different timepoints over the course of tumour progression. These three clusters, identified by single cell sequencing of CD31^-^ stromal cells of murine melanoma tumours sampled at different time points, differed in combined expression of mesenchymal markers and pathways indicating their function including cytokine, chemokines, complement and genes regulating ECM. All three populations were present in all timepoints, however dynamic differences were observed regarding the dominant stromal cluster. Cluster S1, described as “immune” stromal cells, were found early in tumour progression and showed high CXCL12 levels suggesting this might be a driver of early CAF T cell interaction during tumour progression. Similarly, S1 had high CD34 expression with recruitment of macrophages *via* direct crosstalk of C3 and C3aR and low expression of αSMA, whereas the αSMA^high^ Cluster S3 dominated later stage tumours and consisted of “contractile” stromal cells with high expression of genes regulating actin (86).

### Targeting CAFs

**Figure 2:**
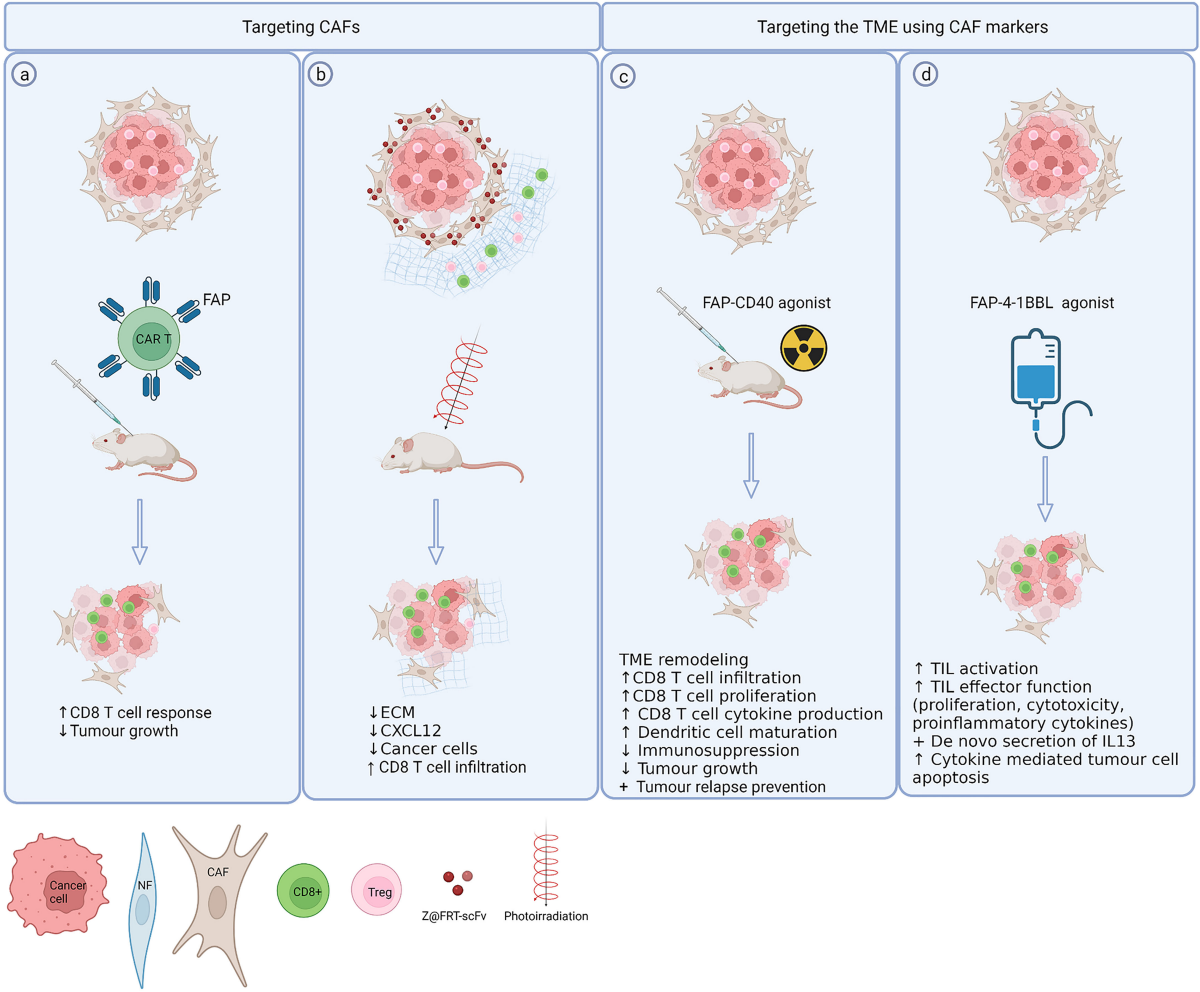
Exploiting CAF markers to selectively target CAFs and the TME. **(A)** Directly targeting CAFs using FAP specific CAR T cells results in reduced tumour growth depending on increased CD8 T cell responses (81, 82). **(B)** A FAP specific single chain variable fragment conjugated to ferritin (Z@FRT-scFv) allows nanoparticle based photoimmunotherapy of CAFs resulting in tumour suppression *via* ECM, CXCL12 reduction and CD8 T cell infiltration (84). **(C, D)** Expression of FAP can be exploited to selectively deliver co-stimulatory agents [CD40 (87), 4-1BBL (88)] to the TME thereby avoiding systemic toxicity. Created with BioRender.com.

Various strategies have been employed to target characteristics of immunosuppressive CAFs such as high FAP expression. (**Figure 2**). CAFs offer a good CAR T cell target as they have powerful multifaceted protumour effects and are more genetically stable than cancer cells. Multiple studies report FAP specific CAR-T cells cause an inhibition of tumour growth in multiple mouse models that was dependent on the immune response (87). and mediated mainly *via* the CD8^+^ T cell response (88) (**Figure 2A**). However, an earlier study by Roberts et al demonstrated that systemic FAP ablation in mice can also have severe adverse effects as it is not only expressed in tumour environments but rather in most tissues of the mouse including skeletal muscle and adipose tissue. Ablation of FAP expressing cells resulted in cachexia and a reduction of erythropoiesis, suggesting that FAP^+^ cells in healthy tissues contribute to essential physiological functions (89). Using a nanoparticle-based photoimmunotherapy method, Zhen et al. conjugated a FAP specific antibody to the nanoparticle ferritin that using photoirradiation allowed local, direct and selective elimination of FAP^+^ CAFs leading to tumour suppression in mice. While this treatment had minimal direct effect on cancer cells, it led to a reduction of serum levels of IL-6 and EGF, known CAF secreted factors and reduced tumour growth was mediated by a reduction of CXCL12 and destruction of ECM (90) (**Figure 2B**).

It is unknown whether targeting CAFs affects their beneficial functions such as TLS formation. Again, CAF heterogeneity must be considered, as TLS formation is driven by FAP^-^ CAFs suggesting that targeting FAP may not interfere with this beneficial function of CAFs (91). It is currently unknown whether depleting FAP affects TLS formation in tumours but a murine model of autoimmune disease demonstrated that genetic depletion of FAP abolishes TLS formation (92).

In addition to inhibiting FAP, the fact that FAP is restricted to the tumour tissue has been exploited to specifically deliver therapies to the TME. As such, FAP has been used as a co-target to deliver the T cell co-stimulatory 4-1BB ligand selectively to the tumour thereby circumventing systemic side effects of 4-1BB ligand such as cytokine release syndrome (93) (**Figure 2D**). Similarly a ligand for a different co-stimulatory receptor CD40 was linked to a bispecific FAP antibody to ensure activation of CD40 was only induced around FAP-expressing cells in an experimental model of murine head and neck cancer that synergised with radiotherapy causing tumour regression and long term survival (94) (**Figure 2C**).

The above evidence suggests that while targeting FAP systemically in mice can have severe adverse effects preclinical studies show that local inhibition of FAP in the TME of tumour bearing mice can be a potent therapeutic tool to allow redistribution of CD8 T cells into the tumour. This illustrates the need for further investigation into underlying mechanisms and suggests that FAP might offer an attractive target to locally modulate immune responses in cancer.

## Conclusion

In conclusion, it has become evident that CAFs significantly impede effective cytotoxic T cell immunity across cancer types. Current knowledge paints the picture that the TME establishes an environment, that promotes the development of hyperactive fibroblasts, CAFs, that perpetually secrete ECM thereby producing a dense web of collagen with high interstitial pressure to protect cancer cells from infiltrating T cells. T cells are trapped in the stroma, physically and *via* chemokines (e.g. CXCL12), CTLA4 and MHC expression, where they are in bidirectional crosstalk with CAFs. This results in reduced proliferation, activation, and differentiation of cytotoxic T cells in an environment that instead nurtures immunosuppressive cells. The reduced number of cytotoxic T cells that do remain are incapacitated by upregulation of co-IR, while their presence causes CAFs to upregulate the corresponding ligands, likely to limit their residual function further.

CAFs affect a multitude of changes in T cell biology, and it seems T cells in turn elicit changes in CAF secretome and surface marker expression. These feedback mechanisms highlight the complex bidirectional crosstalk between CAFs and TILs, illustrating the need for further investigation. However, variable definitions of CAFs and their subtypes remain a challenge in the field as they cause a lack of comparability between studies. Increased understanding of CAF subtypes and whether they are found across cancer types is key to disentangle CAF – TIL interactions. The significant role of CAFs in all steps of the tumour immunity cycle as well as the fact that CAF inhibition resulting in delayed tumour growth was entirely dependent on CTL, underscore that CAFs might present a target to relieve environmental pressures on cytotoxic T cells to increase the efficacy of therapies aimed to revive the cytotoxic T cell response.

## Author Contributions

LK and LM drafted the initial version of the review. All authors edited and revised the review and approved the final manuscript.

## Funding

AA is funded by a Cancer Research UK Clinician Scientist Fellowship (A24867). LM is funded through a EPSRC and MRC Centre for Doctoral Training in Optical Medical Imaging (EP/L016559/1). LK is supported by a GlaxoSmithKline-NPL studentship. The funders were not involved in the review design, interpretation or the writing of this article or the decision to submit it for publication.

## Conflict of Interest

The authors declare that the research was conducted in the absence of any commercial or financial relationships that could be construed as a potential conflict of interest.

## Publisher’s Note

All claims expressed in this article are solely those of the authors and do not necessarily represent those of their affiliated organizations, or those of the publisher, the editors and the reviewers. Any product that may be evaluated in this article, or claim that may be made by its manufacturer, is not guaranteed or endorsed by the publisher.
